# Pulmonary hypertension in systemic sclerosis: diagnosis by systematic screening and prognosis after three years follow-up

**DOI:** 10.1186/s12890-021-01618-z

**Published:** 2021-07-29

**Authors:** Verônica Silva Vilela, Marcio Macri Dias, Ângelo Antunes Salgado, Bruno Rangel Antunes da Silva, Agnaldo José Lopes, Elizabeth Jauhar Cardoso Bessa, Leonardo Palermo Bruno, Cláudia Henrique da Costa, Roger Abramino Levy, Rogério Rufino

**Affiliations:** 1 Rheumatology Discipline, State University of Rio de Janeiro, Hospital Universitário Pedro Ernesto, Third Floor, Boulevard 28 de Setembro 77, Rio de Janeiro, 20551-031 Brazil; 2 Cardiology Discipline, Thorax Diseases Department, State University of Rio de Janeiro, Hospital Universitário Pedro Ernesto, Third Floor, Boulevard 28 de Setembro 77, Rio de Janeiro, 20551-031 Brazil; 3 Immunology and Specialty Medicine, GSK, 1250 Collegville Rd, Collegeville, PA 19426 USA

## Abstract

**Background:**

Systemic sclerosis (SSc) is a rare disease, and the presence of pulmonary hypertension can be a determining factor in prognosis. The aim of this study was to evaluate the diagnosis, profile, and prognosis of systemic sclerosis pulmonary hypertension (SSc-PH) diagnosed by systematic screening in a Brazilian population.

**Methods:**

A cohort of SSc patients underwent systematic screening for SSc-PH. Patients were referred for right heart catheterization (RHC) according to transthoracic echocardiogram or a combination of diagnostic tools. The clinical, immunological, and hemodynamic features and prognosis after 3 years were evaluated.

**Results:**

Twenty patients underwent RHC. SSc pulmonary arterial hypertension (SSc-PAH) was the most common group of SSc-PH. These patients had long disease duration, high urate levels and highly elevated mean pulmonary arterial pressure (mPAP) and peripheral vascular resistance (PVR) on hemodynamics. Patients with mPAP > 20– < 25 mmHg had hemodynamic features of intermediate disease. Patients with SSc-PH associated to interstitial lung disease (SSc-ILD-PH) had signs of vasculopathy on hemodynamics. In patients with no-SSc-PH, the survival at 1, 2, and 3 years was 96%, 92% and 92%, respectively and in patients with SSc-PH it was 86.7%, 60% and 53.3%, respectively.

**Conclusions:**

Patients identified with SSc-PAH and SSc-ILD-PH in our screening had severe clinical and hemodynamic features. Mortality remains high in SSc-PH but was more related to Bo-PAH and SSc-ILD-PH, while in SSc-PAH, the prognosis was better. Trial registration: Current Controlled Trials ISRCTN 72968188, July 8th, 2021. Retrospectively registered.

**Supplementary Information:**

The online version contains supplementary material available at 10.1186/s12890-021-01618-z.

## Background

Systemic sclerosis (SSc) is an autoimmune disease characterized by the concurrent presence of generalized vasculopathy and tissue fibrosis [1, 2]. Effects on the pulmonary system such as fibrosis or pulmonary hypertension (PH) are currently the main cause of death [3]. PH was previously defined as a mean pulmonary artery pressure (mPAP) of ≥ 25 mmHg by right heart catheterization (RHC) [4]. Following which mPAP between 21 and 24 mmHg was considered as “borderline pulmonary artery hypertension” (Bo-PAH) [5]. At the 6th World Symposium of Pulmonary Hypertension, PH was finally defined as mPAP > 20 mmHg with peripheral vascular resistance (PVR) ≥ 3 Woods Units (WU) [6]. The presence of PH in SSc (SSc-PH) can result from vasoclusive pulmonary artery hypertension (SSc-PAH), left ventricular heart dysfunction or pulmonary hypoxic disease, classified as group 1, 2 and 3 PH, respectively [6].

Over the last decade, the development of systematic algorithms for early diagnosis and the data from the follow-up cohorts of incidental SSc-PAH have changed the understanding of this condition. The multiple tools DETECT algorithm, the forced vital capacity (FVC)/ diffusion capacity for carbon monoxide (DLco) ratio or N-terminal- pro- brain natriuretic peptide (NT-pro-BNP) and transthoracic echocardiography (TTE) performed by experts were all proven to achieve early diagnosis of SSc-PAH [7–10]. Furthermore, the reproducibility of the DETECT algorithm was shown in a Czech Republic population using alternative TTE measurements and serum urate levels as the biomarker and achieved early SSc-PAH diagnosis [11]. This provided evidence that some adaption was possible according to diagnostic tests availability. However, there is limited data on such screens on a non-Caucasian and Latin American population.

We performed the present study with the objectives to: (1) evaluate the performance of a systematic screening procedure for SSc-PH diagnosis in a Brazilian population; (2) evaluate the clinical, immunological, and hemodynamic profile of SS-PAH patients diagnosed by this procedure and (3) to evaluate the prognosis of these patients after a three-year of follow-up.

## Methods

### Study population

Patients with systemic sclerosis, according to the American College Rheumatology (ACR)/ European League Against Rheumatism (EULAR) 2013 criteria, treated at our Rheumatology and Pulmonology departments from July 2014 to January 2017, were invited to participate in a systematic screening procedure for RHC referral and early SS-PH diagnosis. The patients screened were unselected SSc patients, e.g. did not show presence of preliminary SSc-PAH risk factors (DLCO < 40% on pulmonary lung function test, severe left heart disease with ejection fraction < 55% on TTE and unwillingness to undergo RHC. Patients with mild to moderate pulmonary disease characterized by FVC or TLC > 40% and ≤ 70% were included due to mixed mechanisms of pulmonary hypertension (SSc-PAH and PH associated to diffuse interstitial lung disease (SSc-ILD-PH)) have been described in patients with this profile. The study was approved by local ethics committee standards and conducted in accordance with the ethical standards laid down in the Helsinki declaration and its latter amendments. All patients signed written informed consent (local ethics committee approval number 314092). The study was registered under the Brazilian Clinical Trials Platform, Brazil Platform CAAE16739013.4.0000.5259 on the March 19th of 2013 (https://plataformabrasil.saude.gov.br/login.jsf) and ISRCTN 72968188.

### Study procedures

The study procedure included (1) systematic SSc-PH screening, (2) RHC in patients that screened positive for SSc-PH, and (3) a second thoracic TTE with estimated mPAP in patients who had undergone RHC.

### SSc-PAH systematic screening

#### Clinical interview and exam

 Demographic and clinical data were collected. SSc was classified according to the LeRoy classification as diffuse cutaneous (dcSSc), in patients with skin thickening proximal to the elbows and knees and involving the trunk, and as limited cutaneous (lcSSc), in patients with skin thickening distal to the elbows and knees and not involving the trunk with or without involvement of the face [12]. The presence of antinuclear antibodies (ANA) and their pattern, the presence of SSc specific autoantibodies antitopoisomerase (anti-Scl-70) and anti-centromere) and antiribonucleoprotein (anti-RNP) were registered in the patient’s information chart. *Blood tests*: Blood samples were collected, and serum urate was used as the PAH biomarker instead of NT-pro-BNP, as it is not widely available in our unit.

#### Pulmonary function tests

Spirometry and DLCO was performed according to the guidelines provided by the America Thoracic Society [13]. TTE: All patients had TTE performed in the past 6 months of the date of the clinical exam and the results from these TTE were used as a referral for RHC according to the European Society of Cardiology and European Respiratory Society 2009 (ESC/ERS) guideline [14]. For patients that underwent RHC, a second TTE was performed to estimate the correlation of mPAP evaluated by the two methods.

A main objective of this study was to identify all cases of SSc-PH. Therefore, we performed the systematic screening by combining diagnostic tools from two sensitive algorithms: the 2009 ESC/ERS guidelines and the DETECT criteria described previously. Patients were referred for RHC if they met one or more of the following two sets of criteria:

1. According to the ESC/ERS 2009 [14]: If tricuspid regurgitation velocity (TRV) was 2.9–3.4 m/s (mPAP 36–50 mmHg) in the absence of symptoms or if TRV was ≤ 2.8 m/second (mPAP ≤ 36 mmHg) and dyspnea of unexplained origin or additional TTE signs of PH are noted.
2. In patients with negative screening according to the criteria 1 and 2, the presence of telangiectasias, anti-centromere, right axis deviation on electrocardiogram, uric acid level, and FVC/DLCO ratio were input into the DETECT calculator step one and when mandated, to step 2 [7].

### Right heart catheterization

During RHC, measurement of mPAP and pulmonary capillary wedge pressure (PCWP) were performed using a Coumard catheter inserted by the Seldinger technique into the right femoral vein. Other hemodynamic measurements performed was: PVR, right atrium (RA) volume, and transpulmonary pressure gradient (TPG). PVR values are expressed by WU, TPG was calculated by subtracting mPAP from PCWP. PH was considered when mean arterial pulmonary pressure was > 20 mmHg on RHC during rest. PH was classified as: group I: vasoclusive or PAH, with mPAP were > 20 mmHg with pulmonary artery wedge pressure (PAWP) ≤ 15 mmHg and pulmonary vascular resistance ≥ 3 Woods Units (WU); group II, due to left heart disease, with mPAP > 20 mmHg and PAWP > 15 mmHg and group III, due to pulmonary hypoxemic disease, with mPAP > 20 mmHg and PCWP < 15 mmHg in the presence of pulmonary disease [6]. SSc-PAH is classified in group PH group I. Additionally, “Borderline pulmonary arterial hypertension (Bo-PAH) was defined in the presence of mPAP > 20– < 25 mmHg. SSc- ILD-PH was diagnosed in patients with mPAP > 20 mmHg and PWAP < 15 in the presence of FVC or TLC < 70% and ≥ 60% and mild to moderate pulmonary interstitial lung disease on high resolution thorax tomography and absence of pulmonary obstructive disease.

### Statistical analysis

Data are presented as mean ± standard deviation for continuous variables and as number (percentage) for categorical variables. The difference in frequency was determined by using chi-square and Fisher’s exact tests. Analysis of variance (ANOVA) was used to compare the distribution of three or more groups in independent samples, with the Tukey test to compare the means. The nonparametric Mann–Whitney test was applied to two independent samples and the agreement analysis between on TTE and RHC by the Bland–Altman method. All analyses were carried out using Stata 14 (StataCorp LP, USA). Kaplan–Meier analysis was used to estimate the survival. Statistical significance was considered when p < 0.05.

## Results

### Study population

Eighty-three SSc patients were consecutively screened from July 2014 to June 2017. Eighteen patients were excluded on baseline screening. Five patients had severe lung disease, 8 had prevalent PH and 5 refused to participate. Sixty-five patients were included; 56 females and 9 males; 25 lcSSc and 40 dcSSc; mean age 50.5, mean disease duration 10 years. Table 1 discloses clinical, laboratorial, pulmonary a TTE features of the entire population. A study database is provided on Additional file 1.

**Table 1:** Demographic, clinical and laboratory data from the 65 included patients

Data	All patients
Age (SD)	50.5 (13.4)
Female (%)	56 (86.1)
Afro-Brazilians (%)	25 (38.4)
Disease duration years (SD)	10.6 (7.7)
Telangiectasia (%)	29 (44.6)
Dyspnea	22 (33%)
Urate levels (SD)	4.5 (1.4)
Left axix deviation	2
FVC % (SD)	77.8 (16.9)
DLco % (SD)	64 (24.1)
FVC/Dlco (SD)	1.3 (0.6)
Detected TRJ (%)	49 (75)
mPAP echocardiography mmHg (SD)	31.9 (14.8)
ANA nucleolar (%)	26 (40)
Antibody positive (%)	27 (41.5)
Anti-Scl-70 (%)	12 (18.4)
Anti-centromere (%)	12 (18.4)

*FVC* forced vital capacity, *Dlco* diffusion capacity for monoxide carbon, *TRJ* tricuspid regurgitation velocity, *mPAP* mean pulmonary arterial pressure, *ANA* antinuclear antibody, anti-Scl-70: anti-scleroderma-70 antibody

### Systematic SSc-PH screening

Sixty-five patients underwent systematic SSc-PH screening. Nineteen patients had TRV > 2.9 and were immediately referred for RHC. Forty-five patients were considered to have low SSc-PAH risk by the three algorithms and were not referred to RHC (low TRJ, and no DETECT criteria). One additional patient had TRV = 2.4 m/second but was referred to RHC by the DETECT criteria (step 1 and 2). A total of 20 patients underwent RHC. These patients underwent a second TTE, performed by the same cardiologist, with expertise in connective tissue disease, within one month after the RHC.

### Right heart catheterization and second TTE

**Fig. 1:**
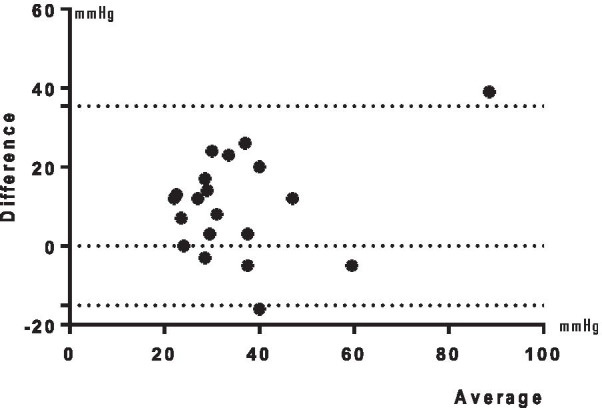
Bland–Altman plot of systolic pulmonary artery pressure by transthoracic echocardiogram and mean pulmonary artery pressure by right heart catheterization. Obs.: In 20 patients, only in 2 (10%) the values were out of 95% limits of agreement (− 15.05 to 35.45)

The 20 patients that underwent RHC were classified as follows: no PH, 5 (25%) patients; SSc-PAH, 11 (55%) patients (among whom 5 had Bo-PAH); SSc-PH group 2, 2 (10%) patients; SSc-PH group 3, 2 (10%) patients. On the second TTE, a high correlation with the mPAP measured in RHC was obtained. Figure 1 discloses the Bland–Altman plot comparing the mPAP in the two methods.

### Clinical and immunological and hemodynamic profile

Table 2 discloses the demographic, clinical and immunological features of the patients with and without SSc-PH. Patients that screened negative (n = 45) plus patients that had no PH on RHC (n = 5) were classified as non-SSc-PH (n = 50). SSc-PH included: SS-ILD-PH, SSc-PH group 2, SSc-PAH and BoPAH. Table 3 discloses the haemodynamic features of the patients that underwent RHC.

**Table 2:** Demographic, clinical, and immunological profile

	Non-SScPH (n = 50)	SSc-ILD-PH (n = 2)	SSc-PH Group 2 (n = 2)	SSc-PAH (n = 11)	SSc-PAH# (Bo-PAH) (n = 5)	P value	Test
Age y–o (SD)	49.27 (14.4)	47 (9.8)	48.5 (12.0)	56.7 (9.7)	61.8 (9.4)	0.03	ANOVA (F = 2.99)
Female (%)	44 (88)	2 (100)	2 (100)	8 (73)	4 (80)	0.16	χ^2^ = 6.54
Afro-Brazilians (%)	15 (30)	2 (100)	2 (100)	6 (54)	3 (60)	< 0.0001	χ^2^ = 54.48
Duration in years (SD)	10.7	5 (0)	7.5 (3.5)	11.7	10.2	0.97	ANOVA (F = 0.07)
lcSSc (%)	20 (40)	0	0	5 (45)	2 (40)	0.82	χ^2^ = 54.48
dcSSc (%)	30 (60)	2 (100)	2 (100)	6 (54)	3 (60)	< 0.0001	χ^2^ = 28.62
Telangiectasia (%)	18 (36)	1 (50)	1 (50)	9 (81)	3 (60)	0.0005	χ^2^ = 20.05
Dyspnea (%)	9 (20)	2 (100)	1 (50)	9 (83)	4 (80)	< 0.0001	χ^2^ = 60.23
Urate mg/dL (SD)	4.37 (1.5)	4.3 (1.7)	4.25 (1.8)	5.58 (1.0)	4.78 (0.5)	0.03	ANOVA (F = 2.99)
Left axis deviation	1	0	0	0	1	0.78	χ^2^ = 0.50
FVC % (SD)	78.0 (17.0)	51.1 (2.8)	75.3 (7.0)	82.0 (13.2)	77.2 (12.4)	0.89	ANOVA (F = 0.20)
DLco% (SD)	71.6 (21.4)	25.6 (0.7)	40.2 (28)	43.7 (14.3)	43.2 (13.2)	< 0.0001	ANOVA (F = 12.91)
FVC/DLco (SD)	1.1 (0.6)	2.1 (0.9)	2.5 (2)	2.0 (0.6)	1.9 (0.4)	< 0.0001	ANOVA (F = 24.02)
Detected TRV (%)	33 (66)	2 (100)	2 (100)	11 (100)	5 (100)	0.04	χ^2^ = 9.92
mPAP on TTE (SD)	24.15 (5.8)	50.5 (50.9)	46.2 (22.3)	40.7 (12.3)	37.6 (10.2)	< 0.0001	ANOVA (F = 10.43)
Nucleolar ANA (%)	18 (36)	1 (50)	2 (100)	4 (36)	2 (40)	< 0.0001	χ^2^ = 56.55
Antibody positive (%)	18 (36)	1 (50)	0	6 (54)	2 (40)	0.19	χ^2^ = 4.71
Anti-Scl-70 positive (%)	9 (18)	1 (50)	0	2 (18)	1 (20)	< 0.0001	χ^2^ = 27.89
Anti-RNP (%)	2 (4)	0	0	1 (9)	0	0.16	χ^2^ = 1.93
Centromere (%)	8 (16)	0	0	3 (27)	1 (20)	0.23	χ^2^ = 2.95
Died (%)	4 (8)	2 (100)	2 (100)	4 (36)	3 (60)	< 0.0001	χ^2^ = 106.52

*lcSSc* limited cutaneous systemic sclerosis, *dcSSc* diffuse cutaneous systemic sclerosis, *Bo-PAH* borderline pulmonary artery hypertension, *FVC* forced vital capacity, *DLco* diffusion capacity for carbon monoxide, *TRV* tricuspid regurgitation velocity, *mPAP* mean pulmonary arterial pressure, *ANA* antinuclear antibody, anti-Scl-70: anti-topoisomerase antibody; anti-RNP: anti-ribonucleoprotein antibody

Obs.: # subgroup of all patients with SSc PAH and mPAP > 20 mmHg—< 25 mmHg, lately named bordeline (Bo-PAH)

**Table 3:** Hemodynamic data

	No-RHC-PH (n = 5)	SSc-ILD-PH (n = 2)	SSc-PH Group 2	SSc-PAH (n = 11)	SSc-PAH# (Bo-PAH) (n = 5)	P value (ANOVA)
mPAP mmHg (SD)	18 (2.00)	48.5 (28.99)	33.0 (4.24)	32.8 (13.31)	22.6 (1.34)	0.03 (F = 3.32)
PAWP mmHg (SD)	11.6 (5.12)	13.5 (2.51)	15.5 (9.19)	13.4 (2.94)	13.2 (3.70)	0.80 (F = 0.41)
PVRi WU (SD)	2.05 (1.04)	34.3 (24.47)	3.11 (0)	6.03 (4.73)	2.66 (0.91)	0.003 (F = 9.26)
RA mmHg (SD)	2.6 (1.51)	8.5 (2.12)	9.0 (0)	10.6 (8.26)	5.4 (1.14)	0.18 (F = 1.72)
TGP mmHg (SD)	6.4 (5.22)	33.0 (19.79)	14.0 (2.82)	27.83 (12.23)	9.4 (3.43)	0.004 (F = 5.990)

*mPAP* mean pulmonary arterial pressure, *PVRi* peripheral vascular resistance index, *PAWP* pulmonary capillary wedge pressure, *RA* right atrium, *SD* standard deviation, *WU* Woods unit, *TGP* transpulmonary pressure gradient

Obs.: # subgroup of all patients with SSc PAH and mPAP > 20 mmHg—< 25 mmHg, lately named bordeline (Bo-PAH)

Patients with SSc-PAH had distinguished features (Table 2). They were frequently lcSSc subtype. There was a higher prevalence of male patients and longer disease duration, more frequent anti-RNP and anti-centromere antibodies in SSc-PAH when compared to the other groups of patients, but this did not reach statistical significance. SSc-PAH patients had significantly more telangiectasia, higher urate levels and FVC%. Most patients were on functional class I or II. Hemodynamics revealed high mPAP, PVRi and TGP, suggesting severe disease (Table 3).

SSc-PH group 2 and SSc-ILD-PH patients had low DLco and high FVC/DLco ratio, were more frequently dcSSc, had shorter disease duration, more frequent nucleolar ANA and anti-Scl antibody. Patients with SSc-ILD-PH had worse functional class and very high PVR and TGP, corroborating concurrent vasoclusive pulmonary arterial and pulmonary parenchymatous disease. Bo-PAH patients were older, had similar percentage of lcSSc and dcSSc, low DLco, high FVC/DLco ratio, had nucleolar ANA, similar percentages of anti-centromere and anti-Scl-70 and high mortality. They were on functional class I or II and had mild increases in mPAP, PVR, RA and TGP.

### Follow-up

**Fig. 2:**
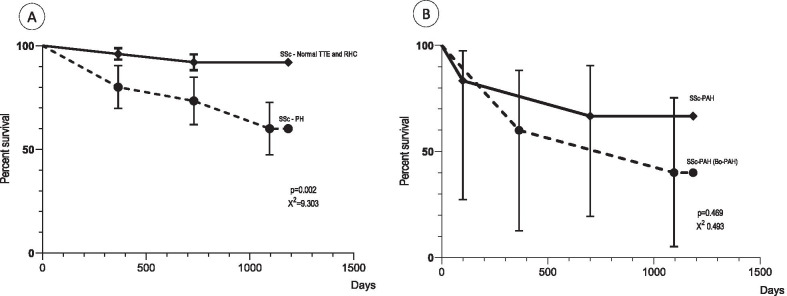
Kaplan Meier survival curve of the patients with and without SSc-PH. Legend: *SSc* Systemic sclerosis, *PH* pulmonary hypertension, *TTE* transthoracic echocardiography, *RHC* right heart catheterization. Obs. **a** SSc-PH had significantly lower survival than patients with non-SSc-PH. **b** patients with SSc-Bo-PAH (former bordeline group) were analyzed separately from other patients with SSc-PAH. These patients had no indication of using specific medications for pulmonary arterial hypertension before 2019

The 65 patients included were followed-up for a mean of 31.5 (SD 7.9) months. Follow-up time commenced at the time of providing informed consent or from the date of the RHC. The end follow-up was at 36 after the inclusion or in April 2019 for patients that were included later. The cause of death was confirmed in hospital files and family reports, the date was confirmed by the hospital files or from the State Registry of Deaths. To analyze mortality, we compared the survival of the patients with SSc-PH and Bo-PAH (SSc-PH group 2, SSc-ILD-PH, SSc-PAH, mPAP > 20– < 25 mmHg, n = 15) to patients considered to have no PH (negative screening plus no-SSc-PH on RHC, n = 50). Figure 2 discloses the Kaplan Meier curve with this result.

In patients who were considered to have no-SSc-PH, the survival rate at one, two and three years was 96%, 92% and 92%, respectively. In the group of patients classified as SSc-PH, survival at one, two and three years was 86.7%, 60% and 53.3%, respectively (Fig. 2a). The evolution, management, and survival, however, was different in each group of SSc-PH. Patients with Bo-PAH had lower survival when compared to the patients with SSc-PH that received treatment (SSc-PH groups II and III and SSc-PAH with mPAP ≥ 25 mmHg) (Fig. 2b).

SSc-PAH patients with mPAP ≥ 25 mmHg received early specific treatment for PAH and tight control, with drugs combination or exchange as rapidly as necessary. They had a chance of survival of 75% at 3 years. One patient received sildenafil 20 mg 3 times/day but had rapidly progressive disease and died 5 months after the RHC. Three patients received initial treatment with sildenafil 20 mg three times/ day. After one year they worsened functional class and mPAP estimated on follow-up TTE and bosentan was added. These three patients remained stable until the end of follow-up with the combination of sildenafil 20 mg three times/day plus bosentan 125 mg two times/day. The two remaining patients also worsened functional class and mPAP (one after six month and one year) of sildenafil 20 mg three times/day. Both did not improve after adding bosentan 125 mg two times/day for six months. They had the combined sildenafil plus bosentan therapy replaced by macitentan 10 mg/ day and had stabilized functional class and mPAP on TTE. Two patients were in use of imunossupressants (mycophenolate), that was not related to the prognosis.

Among patients with Bo-PAH, no patients received PAH specific therapy; 3 (60%) died: 2 with unexpected rapidly progressive dyspnea and 1 with multiple organs involvement. From the surviving patients, one developed rapidly progressive SSc-PAH confirmed on a second RHC one year later and the other remained stable. Two patients classified as SSc-ILD-PH died from progressive ILD. Two patients with SSc-PH group 2 died due to cancer (1 lymphoma and 1 lung cancer). All patients with SSc-ILD-PH and SSc-PH group 2 were in use of immunossupressants: 1, rituximab; 1, mycophenolate and 2, azathioprine. Among the patients Bo-PAH, one patient received prednisone (20 mg/day) and one received mycophenolate, that did not interfere with the prognosis.

## Discussion

In this study, we performed a systematic screening for SSc-PH, evaluated the clinical, immunological, and hemodynamic profile and prognosis of these patients. We found that SSc-PAH was the most frequent in this sample and had severe hemodynamic features. Patients with SSc-PH group II and SSc-ILD-PH were also identified. Finally, survival in different groups of patients was evaluated.

After the screening, TTE identified most patients with SSc-PH according to the previous criteria. However, only with the DETECT algorithm one additional patient with mPAP > 20– < 25 mmHg could be referred to RHC. In a previous large cohort, TTE could identify all patients with mPAP ≥ 25 mmHg but adding to DETECT tools was necessary to the identification of with Bo-PAH patients [15]. Thus, the 2009 ESC/ERS guidelines for RHC referral is suitable to diagnose patients with mPAP ≥ 25 mmHg, but more sensitive referral criteria are necessary to identify patients with mPAP > 20– < 25 mmHg. The good correlation between mPAP on RHC and TTE can be achieved when TTE is performed by echocardiographis experienced on SSc [8].

SSc-PAH was the most common group of PH. The age at onset of SSc-PAH was 56.7 years old, similar with non-European ancestry cohorts [19–21]. Anywhere SSc-PAH patients had high urate levels. This was previously described only in the DETECT study and in the Hopkins Cohort [7, 16]. The high frequency of nucleolar ANA in SSc-PAH has been previous 265 reported in the study by Steen et al. and in the PHAROS cohort [17, 18]. Since this ANA 266 pattern is related to new SSc specific autoantibodies (anti-Th/To and anti-fibrillarin), we corroborate the correlation between these antibodies and the increased risk of SSc-PAH. This was in accordance with previous studies that had revealed that non-European populations had worse functional class and hemodynamic profile than European ancestry [19–22].

We could also identify patients with group 2 SSc-PH and SSc-ILD-PH. Our patients with SSc-ILD-PH had very high PVR and mPAP. Young et al. [23] previously found that combined SSc-ILD-PH is common and that this group of patients had hemodynamic features of SSc-PAH. This evidence suggests that SSc-ILD-PH frequently results from combined pulmonary artery vasculopathy and intrinsic pulmonary parenchymal disease. In SSc, pulmonary arterial vasoclusive disease, left heart fibrosis and ILD occur at the same time. In the PHAROS cohort SSc-PH classification changed in 30% of the patients that underwent a second RHC [24]. RHC results represents the static measures of a dynamic disease and should be interpreted with caution.

Our patients with mPAP > 21– < 25 mmHg had a demographic, clinical and immunological profile with mixed features of SSc-PAH and SSc-ILD-PH. Functional class and hemodynamic profile were intermediate between SSc-PH and SSc-PAH, also with an increased TPG. This is in accordance with the DETECT post-hoc study, were patients with mPAP > 1– < 25 mmHg were considered as a group with an undefined profile, with features both heart, lung, and pulmonary arterial disease and frequently an increased TPG [25].

Patients with SSc-PH and Bo-PAH had significantly higher mortality than patients with no PH. Thus, PH is a major cause and condition related to death in SSc patients. The evolution, management, and survival, however, was different in each group of SSc-PH or in patients with Bo-PAH.

SSc-PAH patients received early specific treatment and tight control. Although the baseline hemodynamic features of our patients were significantly severe, their prognosis was relatively good. Similar with the reported in the long-term analyzes of the PHAROS cohort, mortality in our patients occurred early in the clinical course of SSc-PAH, and the survival at 3 years was 75% among the patients that received specific treatment [26]. This was significantly better than 55% survival at 3 years achieved in past studies [27]. Thus, systematic screening and early diagnosis may improve the survival in patients with SSc-PAH. It is possible that the early and aggressive therapy might have improved the survival in these patients.

Patients with Bo-PAH mmHg had 60% survival at 3 years. We speculate that if these patients had been diagnosed and treated as SSc-PH, their survival could have been better. Two previous studies, the follow-up of the PHAROS cohort and the single center study of Valerio et al. [5], also disclosed that a significant percentage of patients with mPAP > 21– < 25 mmHg evolve to mPAP ≥ 25 mmHg and have bad prognosis. These results corroborate that mPAP > 20– < 25 mmHg is not a benign condition and endorses the new 6th World Symposium Classification, that determines that PH should be diagnosed with a lower mPAP threshold of > 20 mmHg [6].

Our patients with SSc-PH group 2 and SSc-ILD-PH had 100% of mortality. Group 2 patients died from cancer. SSc-PH may be an associated condition that adds morbidity rather than being the specific cause of death. Regarding SSc-ILD-PH, our patients also had a 100% mortality at 3 years. Due to the coexistence of pulmonary arterial vasculopathy and pulmonary parenchymal vessels destruction, the presence of PH significantly increases the mortality of SSc-ILD. It was previously disclosed that specific medications for pulmonary arterial hypertension (sildenafil, bosentan and prostanoids) had no beneficial effect in the treatment of pulmonary hypertension related to ILD [28].

This study has several limitations. The number of patients recruited achieved the sample size calculated but some patients had exclusion criteria and did not complete the study. We did not perform RHC for all patients included in the screening and it is not possible to no be sure that no SSc-PH patient were missed. As symptomatic patients were included, it could be argued that this was not a completely inclusion cohort. However, we thought that it was important to include these patients since they were mainly in functional class I or II, suggesting early disease. A strength is that by the longitudinal design, we could evaluate the survival.

## Conclusion

A systematic procedure to screen SSc-PH disclosed patients with early but severe disease, frequently with specific autoantibodies and with high mortality. TTE could identify all patients with mPAP ≥ 25 mmHg but more sensitive criteria were necessary to detect mPAP > 20 mmHg. Survival was significantly lower in patients with SSc-PH than in SSc with no PH. However, we found a different pattern of mortality among our patients with SSc-PH. In previous studies, mortality in SSc-PH was related to the severity and 331 lack of treatment in SSc-PAH. In our study, high mortality was related to the absence of treatment among patients with Bo-PAH, to the impact of PH as a comorbidity in SSc patient and as coexistent vasculopathy increasing the severity of SSc-ILD.

## Supplementary Information

**Additional file 1.**: The results of clinical, laboratory, spirometric, echocardiographic and hemodynamic data.

## Data Availability

The datasets generated and/or analyzed during the current study are available on the Additional file 1. The main author (Verônica Silva Vilela, e-mail: vilelavs@gmail.com) is the person to be contacted for access to data.

## Abbreviations

ACR: American College Rheumatology
ANA: Antinuclear antibodies
Anti-RNP: Antiribonucleoprotein
ASIG: Australian Scleroderma Interest Group
Bo-PAH: Borderline pulmonary artery hypertension
dcSSc: Diffuse cutaneous systemic sclerosis
DLco: Diffusion capacity for carbon monoxide
EULAR: European League Against Rheumatism
FVC: Forced vital capacity
lcSSc: Limited cutaneous systemic sclerosis
mPAP: Mean pulmonary arterial pressure
NT-pro-BNP: N-terminal- pro-brain natriuretic peptide
PAH: Vasoclusive pulmonary arterial hypertension
PCWP: Pulmonary capillary wedge pressure
PH: Pulmonary hypertension
PVR: Peripheral vascular resistance
PVRi: Periferal vascular resistance index
RA: Right atrium
RHC: Right heart catheterization
SD: Standard deviation
SSc: Systemic sclerosis
TGP: Transpulmonary pressure gradient
TLC: Total lung capacity
TRV: Tricuspid regurgitation velocity
TTE: Transthoracic echocardiography
WU: Woods units

## References

[1] Denton CP, Khanna D (2017). Systemic sclerosis. Lancet.

[2] Denton CP, Wells AU, Coghlan JG (2018). Major lung complications of systemic sclerosis. Nat Rev Rheumatol.

[3] Elhai M, Meune C, Boubaya M, Avouac J, Hachulla E, Balbir-Gurman A (2017). Mapping and predicting mortality from systemic sclerosis. Ann Rheum Dis.

[4] Sahay S (2019). Evaluation and classification of pulmonary arterial hypertension. J Thorac Dis.

[5] Valerio CJ, Schreiber BE, Handler CE, Denton CP, Coghlanet JG (2013). Borderline mean arterial pressure in patients with systemic sclerosis: transpulmonary gradient predicts risk of developing pulmonary hypertension. Arthritis Rheum.

[6] Simonneaul G, Montani D, Celermajer DS, Denton CP, Gatzoulis MA, Krowka M (2019). Haemodynamic definitions and updated clinical classification of pulmonary hypertension. Eur Respir.

[7] Coghlan JG, Denton CP, Grunig E, Bonderman D, Distler O, Khanna D (2014). Evidence based pulmonary arterial hypertension in systemic sclerosis: the DETECT study. Ann Rheum Dis.

[8] Hachulla E, Gressin V, Guillevin L, Carpentier P, Diot E, Sibilia J (2005). Early 408 detection of pulmonary arterial hypertension in systemic sclerosis: a French national prospective study. Ann Rheum Dis.

[9] Hao J, Thakkar V, Stevens W, Morrisroe K, Prior D, Rabusa C (2015). Comparison of the predictive value of three screening models for pulmonary arterial hypertension in systemic sclerosis. Arthritis Res Ther.

[10] Hachulla E, de Groote P, Gressin V, Sibilia J, Diot E, Carpentier P (2009). Three-year incidence of pulmonary arterial hypertension associated with systemic sclerosis in a multicenter nationwide longitudinal study in France. Arthritis Rheum.

[11] Soukup T, Pudil R, Kubinova K, Hromadkova L, Dusek J, Tosovsky M (2016). Application of the DETECT algorithm for detection of risk pulmonary artery hypertension in systemic sclerosis: data from a Czech tertiary center. Rheumatology.

[12] Herrick AL, Hughes M (2019). Systemic sclerosis. Br J Hosp Med Lond.

[13] Culver BH, Graham BL, Coates AL, Wahger J, Berry CE, Clarke PK (2017). ATS Committee on Proficiency Standards for Pulmonary Function Laboratories. Recommendations for a standardized pulmonary function report. An official American Thoracic Society technical statement. Am J Respir Crit Care Med.

[14] Galiè N, Hoeper MM, Humbert M, Torbicki A, Vachiery L (2009). Guidelines for the diagnosis and treatment of pulmonary hypertension: The Task Force for the Diagnosis and Treatment of Pulmonary Hypertension of the European Society of Cardiology (ESC) and the European Respiratory Society (ERS), endorsed by the International Society of Heart and Lung Transplantation (ISHLT). Eur Heart J.

[15] Vandercasteele E, Drieghe B, Melsens K, Thevissen K, De Pauw M, Deschepper E (2017). Screening for pulmonary artery hypertension in an unselected prospective systemic sclerosis cohort. Eur Resp J.

[16] Simpson CE, Damico RL, Hummers L, Khair RM, Kolb TM, Hassoun PM (2019). Serum uric acid as a marker of disease risk, severity, and survival in systemic sclerosis related pulmonary arterial hypertension. Pulm Circ.

[17] Steen VD, Lucas M, Fertig N, Medsger TA (2007). Pulmonary arterial hypertension and severe pulmonary fibrosis in systemic sclerosis patients with a nucleolar antibody. J Rheum.

[18] Chung L, Domsic RT, Lingala B, Alkassab F, Bolster M, Csuka ME (2014). Survival and predictors of mortality in systemic sclerosis-associated pulmonary arterial hypertension: outcomes from the pulmonary hypertension assessment and recognition of outcomes in scleroderma registry. Arthritis Care Res.

[19] Beall A, Nietert P, Taylor MH, Mitchell HC, Shaftman SR, Silver RM (2007). Ethnic disparities among patients with pulmonary hypertension associated with systemic sclerosis. J Rheum.

[20] Blanco I, Mathai S, Shafiq M, Boyce Kolb TM, Chami H, Hummers LK (2014). Severity of systemic sclerosis-associated pulmonary arterial hypertension in African 451 Americans. Medicine.

[21] Al Otair HAK, Idress MM, Saleemi SA, Eltoukhy AM, Alhijii AA, Habeeb WAA (2019). Pulmonary arterial pression in Saudi patients with systemic sclerosis: clinical and hemodynamic characteristics and mortality. Ann Thorac Med.

[22] Mendes C, Viana VST, Pasoto SG, Leon EP, Bonfa E, Sampaio-Barros PD (2020). Clinical and laboratory features of African-Brazilian patients with systemic sclerosis. Clin Rheum.

[23] Young A, Vummidi D, Visovatti S, Homer K, Wilhalme H, White ES (2019). Prevalence, treatment, and outcomes of coexistent pulmonary hypertension and interstitial lung disease in systemic sclerosis. Arthritis Rheum.

[24] Lammil MR, Ann Saketkoo L, Gordon JK, Steen VD (2018). Changes in hemodynamic classification over time are common in systemic sclerosis-associated pulmonary hypertension: insights from the PHAROS cohort. Pulm Circ.

[25] Visovatti SH, Distler O, Coghlan JG, Denton CP, Grünig E, Bonderman D (2014). Borderline Pulmonary artery hypertension in systemic sclerosis patients: a post-hoc analysis of the DETECT study. Arthritis Res Ther.

[26] Kolstad KD, Li S, Chung L, PHAROS investigators. Long-Term Outcomes in Systemic Sclerosis-Associated Pulmonary Arterial Hypertension from the Pulmonary Hypertension Assessment and Recognition of Outcomes in Scleroderma Registry (PHAROS). Chest. 2018;154(4):862–71. 10.1016/j.chest.2018.05.002. 10.1016/j.chest.2018.05.002 PMC6207791 29777655

[27] Chung L, Farber HW, Benza R, Miller DP, Parsons L, Hassoun PM (2014). Unique predictors of mortality in patients with pulmonary arterial hypertension associated with systemic sclerosis in the REVEAL Registry. Chest.

[28] LePavec J, Girgis RE, Lechzin N, Mathai SC, Launay D, Hummers LK (2011). Systemic sclerosis-related pulmonary hypertension associated with interstitial lung disease: impact of pulmonary arterial hypertension therapies. Arthritis Rheum.

